# First Characterisation of Volatile Organic Compounds Emitted by Banana Plants

**DOI:** 10.1038/srep46400

**Published:** 2017-05-16

**Authors:** Chadi Berhal, Caroline De Clerck, Marie-Laure Fauconnier, Carolina Levicek, Antoine Boullis, Amine Kaddes, Haïssam M. Jijakli, François Verheggen, Sébastien Massart

**Affiliations:** 1 Laboratory of Integrated and Urban Phytopathology, Gembloux Agro-Bio Tech, University of Liège, Liège, Belgium; 2 Laboratory of General and Organic Chemistry, Gembloux Agro-Bio Tech, University of Liège, Liège, Belgium; 3 Laboratory of Functional and Evolutionary Entomology, Gembloux Agro-Bio Tech, University of Liège, Liège, Belgium.

## Abstract

Banana (*Musa sp*.) ranks fourth in term of worldwide fruit production, and has economical and nutritional key values. The Cavendish cultivars correspond to more than 90% of the production of dessert banana while cooking cultivars are widely consumed locally around the banana belt production area. Many plants, if not all, produce Volatile Organic Compounds (VOCs) as a means of communication with their environment. Although flower and fruit VOCs have been studied for banana, the VOCs produced by the plant have never been identified despite their importance in plant health and development. A volatile collection methodology was optimized to improve the sensitivity and reproducibility of VOCs analysis from banana plants. We have identified 11 VOCs for the Cavendish, mainly (E,E)-α-farnesene (87.90 ± 11.28 ng/μl), methyl salicylate (33.82 ± 14.29) and 6-methyl-5-hepten-2-one (29.60 ± 11.66), and 14 VOCs for the Pacific Plantain cultivar, mainly (Z,E)-α-farnesene (799.64 ± 503.15), (E,E)-α-farnesene (571.24 ± 381.70) and (E) β ocimene (241.76 ± 158.49). This exploratory study paves the way for an in-depth characterisation of VOCs emitted by *Musa* plants.

Classified 4^th^ in terms of fruit production in the developing countries, the banana plant (*Musa sp.*) is cultivated in 130 countries, occupying more than 10.5 Million ha in the Banana belt^1^. The 143 million tons produced worldwide fall in two categories: the dessert varieties, commonly consumed as a fruit, and called “Banana”, and the cooked/fried varieties, consumed as a vegetable, and commonly called “Plantain”. The former is better known in the American and European trade markets, while the later is more popular in African and Asian countries^2^,^3^. The Cavendish cultivar group is the most appreciated and cultivated one^4^, offering sweeter, bigger and softer free seeded pulp among other varieties. The origin of this popular cultivar group is the diploid *Musa acuminata* AA (2n = 22), giving seedless fruits through parthenocarpy. Due to mediated polyploidization process, the present cultivar group fall in the AAA (3n = 33) or AAAA (2n = 44) subgroups, offering bigger and sweeter fruits than their ancestors^3^. While the Cavendish cultivar group alone is responsible for 40% of the produced banana, the plantain (a triploid AAB) is responsible for 21% of the production, falling 2^nd^ in importance behind Cavendish^4^.

Plants communicate with their environment through signals. Volatile Organic Compounds (VOCs) are considered key elements in this interaction^5^,^6^. They are airborne, high vapour pressured secondary metabolites, documented to be involved in attracting pollinators and seed dispersers, and in preventing attacks of herbivores and pathogens^7^,^8^. The VOCs can be considered as a language for the plants, allowing plant-to-plant or plant-environment communications. The study of the VOCs is gaining an increasing interest in the scientific community due to their importance^8^,^9^.

Even if the VOCs emitted by banana fruits and flowers were characterized by Facundo *et al*.^10^ and Bestmann *et al*.^11^ respectively, VOCs emitted by the banana plant itself were never studied. Therefore, this study aims to characterize for the first time the VOCs produced by a representative accession of two important categories of banana: Cavendish (AAA) and Pacific Plantain (AAB).

## Results

### Adaptation of VOCs collection and analysis protocol

The development of a reliable and sensitive protocol was a challenge due to the low level of VOCs emission by the banana plant. Several steps of the extraction protocol (sampling method, sampling time) were optimized compared to existing protocols^12^ and the improved protocol is described in the material and methods section.

We have robustly identified 11 compounds emitted by Cavendish plants and 14 emitted by Pacific Plantain (Table 1). The selection criteria and validation of the VOCs extracted for each representative variety were: their presence in at least 3 of 4 repetitions, and the difference in emissions or total absence in the control sample. The identification of the compounds was through their Mass Spectrum (MS) and their Retention Index (RI). Most of these compounds belong to the terpenes group (8 for Cavendish, 10 for Pacific Plantain). The other compounds detected were ketones, esters and aldehydes.

Eight compounds were common between the two varieties (myrcene, Z and E β-ocimene, 6-methyl-5-hepten-2-one, 6-methyl-3,5-heptadien-2-one, α-farnesene, methyl salicylate and β-ionone). The main compounds emitted by Cavendish and Pacific Plantain cultivars are (E,E)-α-farnesene (87.9 ± 11.3 ng/μl) and (Z,E)-α-farnesene (799.6 ± 503.1 ng/μl), respectively.

## Discussion

**Figure 1:**
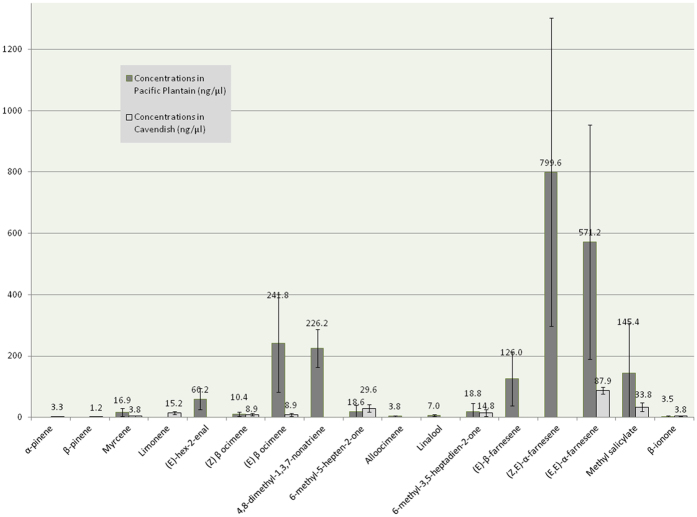
Quantities of volatile organic compounds produced by Cavendish (light grey bars), and Pacific Plantain (darker bars).

We have determined that eight VOCs were emitted by both Cavendish and Pacific Plantain varieties, representing a relative proportion of 73 and 57% of the total emitted VOCs for Cavendish and Pacific Plantain respectively. Among these, three VOCs ((Z) β ocimene, 6-methyl-3,5-heptadien-2-one and β-ionone) were produced in similar proportions between the two varieties. The relative proportions among the remaining common VOCs (Fig. 1) were mostly higher on the side of the Pacific Plantain compared to Cavendish (4, 27, 6.5 and 4 times higher for myrcene, (E) β ocimene, (E,E)-α-farnesene and methyl salicylate respectively). The only exception was 6-methyl-5-hepten-2-one, which was 1.5 times higher in Cavendish than in Pacific Plantain. Limonene, α- and β-pinene were only detected with Cavendish while Pacific Plantain emitted specifically (E)-hex-2-enal, DMNT, (E)-β-farnesene, linalool and alloocimene.

An extensive bibliographical analysis was carried out on the 17 VOCs identified in this study. A first comparison has been made with the VOCs known to be produced by other banana organs (see Table 2). Among the VOCs identified in this study, four were already detected in *Musa sp*. flowers (α and β-pinene, limonene and myrcene)^11^, three in the whole fruit of *M. acuminata* cv. Nanicão (α-pinene, limonene, and ocimene), and two in the pulp of the aforementioned cultivar (limonene, (E)-hex-2-enal)^10^. Limonene is therefore the only VOC detected in the four studies related to *Musa* genus. It was however only found on Cavendish plants in our study.

In addition, VOCs were compared to the identified compounds of other plants in the same Order (Zingiberales), and in particular to the ones emitted by the flowers of *Hedychium coronarium*, commonly named “Ginger lily”^13^. Fourteen VOCs (Table 2) were identified in these studies. We observed that three of them (limonene and α and β-pinene) were specifically emitted by Cavendish, four were specific to Pacific Plantain ((E)-hex-2-enal, DMNT, alloocimene and linalool) and seven were common between the two varieties. Furthermore, all the 17 VOCs detected in our work have already been identified in other plants^14^ (Table 2), but to our knowledge, the blend of VOCs emitted by Pacific Plantain and Cavendish varieties might be unique, in confirmation of the general likelihood of a taxa-specific VOCs emission^15^,^16^. This hypothesis would nevertheless need the additional testing of many other varieties and closely related species.

According to Pherobase and a bibliographical survey^14^, all 17 identified VOCs are known to play a role in insects control, and 11 identified VOCs (7 for Cavendish (64% of the total emission) and 8 for Pacific Plantain (57%)) have a role in controlling plant diseases. Among all identified VOCs, and regarding their functions in insect management, 14 showed an attraction function (91% for Cavendish and 79% for Pacific Plantain), 13 are allomones (82% for Cavendish and 71% for Pacific Plantain), 16 are kairomones (91% for Cavendish and 93% for Pacific Plantain), and 15 are pheromones (100% for Cavendish and 86% for Pacific Plantain). It is worth mentioned that methyl salicylate (second most abundant compound emitted by Cavendish and fifth by Pacific Plantain) is also known to be an herbivore induced compound, that the plant used as an insect protection agent^17^.

In addition, 11 VOCs have also shown antimicrobial effects. Alpha and β-pinene have antimicrobial activities^18^,^19^. Myrcene is known for its antifungal^20^ and antibacterial properties^21^. Limonene presents a strong antifungal activity^20^,^22^,^23^,^24^ and it has been proven that antifungal activity of citrus essential oils on *Aspergillus flavus, Penicillium chrysogenum* and *Penicillium verrucosum* was explained by its prevalence^25^.

The (E)-hex-2-enal, belonging to the Green Leaf Volatiles (GLVs) group, is well known to exhibit an antimicrobial activity^26^ and a strong antifungal activity *in-vitro*^27^, and in fields of soybean, conference pears, stone fruits, and common bean^28^. This VOC is also implicated as a defence response mechanism in the Lima bean^29^, similarly to *Nicotiana tabaccum*^30^ when infected. Another defence signalling compound is the (E)-β-ocimene^31^,^32^.

Herman *et al*.^33^ found that linalool significantly increased the antimicrobial activity of essential oils when it was combined to them, and suggest that the synergism could be through enhancing the uptake of other antimicrobials by the cell wall of the pathogens^33^. In addition, methyl salicylate, which is the main compound of the essential oils of *Laportea aestuans*, showed inhibitory potential against *E. coli* and *S. aureus*^34^. At the opposite, 6-methyl-5-hepten-2-one (third most abundant in Cavendish and ninth in Pacific Plantain) was found to be an endogenous germination stimulators of uredospores of *Puccinia graminis* var. *tritici*^35^.

All the compounds emitted specifically by Pacific Plantain show protection properties. (E)-hex-2-enal, DMNT^36^ and linalool^37^ have insect and disease control properties. (E)-β-farnesene, is an alarm pheromone emitted by aphids in case of predator or parasitoid attack^38^, that can be used by plants as repulsive agent^39^,^40^. Alloocimene^41^ and linalool^42^ also showed disease control properties.

In addition, the GLV (E)-hex-2-enal, and methyl salicylate, are reported to be involved in the plant-plant communication, as an alarm signal for the neighbouring plants, when they are exposed to injured plants VOCs^8^.

This study opens therefore the field on new research axes to further investigate if several Cavendish and/or Pacific Plantain VOCs identified, are involved, individually or in a blend with specific concentration ratios, in plant-pathogen and plant-plant interaction.

## Conclusion

This study reports for the first time the VOCs emitted by aerial parts of Cavendish and Pacific Plantain banana plants. Despite low levels of emission, the VOCs collection revealed that Cavendish emitted 11 compoundsVOCs, mainly (E,E)-α-farnesene (87.90 ± 11.28 ng/μl), methyl salicylate (33.82 ± 14.29) and 6-methyl-5-hepten-2-one (29.60 ± 11.66), while Pacific Plantain emitted 14 VOCs, mainly (Z,E)-α-farnesene (799.64 ± 503.15), (E,E)-α-farnesene (571.24 ± 381.70) and (E) β ocimene (241.76 ± 158.49). Even if the majority of the detected compounds (11 of all the VOCs combined) have known disease and insect control properties, they are produced at low levels and their potential effect in *Musa* should be further confirmed. In conclusion, this exploratory study paves the way for an in-depth characterisation of VOCs emitted by the Cavendish and Pacific Plantain, focusing on their roles and emissions in biotic and abiotic stress management.

## Materials and Methods

### Banana plants

The plant material used was the banana cultivar Williams, of the Cavendish cultivar group (AAA genome), and the banana cultivar Pacific Plantain, of the Maia Maoli/Popoulu cultivar group (AAB genome), grown from *in vitro* meristems, kindly provided by the International Transit Center (ITC) for banana germplasm (Bioversity, Leuven, Belgium). Growing conditions in greenhouse were 25 ± 2 °C at 16 h/8 h (Day/Night) photoperiod. The plants were grown in pots of 20 cm diameter, containing a peat moss substrate, and irrigated every 1 or 2 days when needed, until they reach 40–60 days of age, stage of VOCs extraction, having 5–6 leaves. The plants submitted to VOCs sampling were virus-free, they had neither visible disease symptoms nor observable pests, and did not present any wound.

### Collection and analysis of VOCs

**Figure 2:**
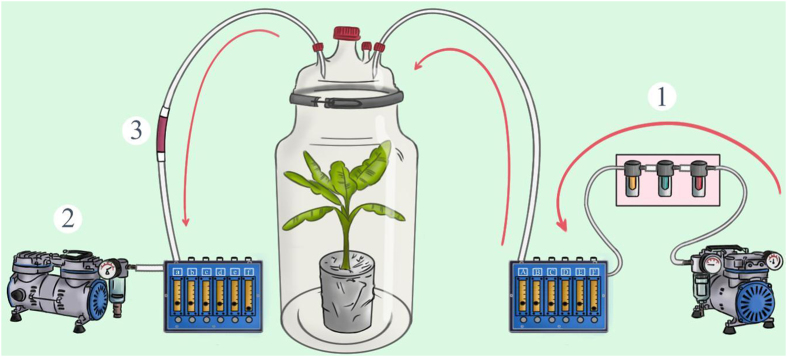
Extraction of VOCs emitted by the Banana plant upper part. The filtered air (1) is pumped in the enclosed Banana environment, after covering its basal part. A second pump (2) ensure the second phase of the “Push-Pull” system applied, leading the enriched air to pass through a trap filter (3), prior to the analysis. (Illustrated by: Carolina LEVICEK, 2015).

The volatile collection protocol was a dynamic system, and a thermal desorption was performed for the analysis method, due to its efficiency compared to a solvent desorption^13^. Four entire banana plants (50 cm high, 5 to 6 leaves) of each variety were placed at each extraction in 4 identical glass chambers (40,000 ml), previously cleaned with RBS detergent (Chemical Products R. Borghgraef S.A., Belgium) and rinsed with distilled water, to collect the volatile compounds, using a “Push-Pull” system (Fig. 2). The air was pushed through the system by an air pump (Rocker 430 Vacuum/Pressure Pump, NSE, GA, USA) that provided a constant airflow set at 1000 ml/min. The air entering the chamber was previously cleaned (Clean Air Supply System CASS 6, Volatile assays, Rensselaer, NY, USA). A filter, made of a glass cartridge filled with 60 mg of adsorbent material (TENAX TA wax trap, Gerstel, Mülheim an der Ruhr, Deutschland), was placed at the exit of the glass chamber and was used to catch the volatile compounds carried by the air being pulled from the chamber using a second air pump (Rocker 400 Vacuum Pump, NSE, GA, USA). The filters were previously cleaned by a thermal conditioner (TC2, Gerstel, Mülheim an der Ruhr, Deutschland), for a period of 11 hours at 300 °C prior to each extraction. Four replicates per variety were conducted simultaneously, along with a fifth chamber containing a pot filled with the same substrate, but without the plant. The VOCs collection was performed at 24 ± 2 °C, with a relative humidity of 47 ± 6% and an artificial photoperiod of 16 h/8 h (Day/Night) under LED lamps (77 lmol/sqm/s).

The collected VOCs were analyzed by a Gas Chromatography-Mass Spectrometry (GC-MS) thermo desorption method (7890A, Agilent Technologies, Santa Clara, CA, USA). In this system, the content of the TENAX TA trap was thermally desorbed (Thermal Desorption Unit, Gerstel, Mülheim an der Ruhr, Deutschland) at 280 °C for 10 minutes prior to the injection. In each sample, 1 μl of an Internal Standard (I.S.) (butylbenzen, 100 ng/ml), diluted from a pure solution (Sigma-Aldrich, Saint-Louis, MO, USA) in a methanol solvent (VWR, West Chester, PA, USA) was automatically added for the quantification control purpose (MultiPurpose Sampler, Gerstel, Mülheim an der Ruhr, Deutschland).

The entire sample was injected in the column (polar column VF-WAXms, 30 m, internal diameter: 0.25 mm, thickness: 0.25 μm, Agilent Technologies, Santa Clara, CA, USA) of the GC machine. Conducted by a Helium gas (1.5 ml/min), the VOCs were prone to the following temperature program: 35 °C for 2 minutes, followed by an increase of 5 °C/min up to 155 °C, and then 20 °C/min until reaching 250 °C, which was maintained for 10 minutes.

The identification that followed was insured by the Mass-Spectrometer (5973, Agilent Technologies, Santa Clara, CA, USA) via the electronic impact ionisation (70 eV), and a separation through the quadripolar filter at 150 °C.

MSD ChemStation Software (Agilent Technologies, Santa Clara, CA, USA) was used to characterize the chromatograms received from all previous analyses, by the mean of manual integration, comparing the retention times with the WILEY275 spectral database, only taking into consideration the compounds found at least in 3 of the 4 repetitions. The compounds were rejected if similar quantities are collected from both the control and the plants. Further identification was carried out by comparing the theoretical Kovats retention indices (RI) with calculated ones for each molecule. RI were calculated by injecting a saturated n-alkane standard solution (C_7_–C_30_) at 1,000 μg/mL in hexane (Supelco, Belgium), following the definition of van Den Dool and Kratz^43^. In addition, identifications were confirmed by injecting commercial standards provided by Sigma-Aldrich (Sigma-Aldrich, Saint-Louis, MO, USA).

Quantifications were determined by constructing calibration curves, for each volatile chemical group, injecting authentic standards in the same previews analysis conditions.

## Additional Information

**How to cite this article:** Berhal, C. *et al*. First Characterisation of Volatile Organic Compounds Emitted by Banana Plants. *Sci. Rep.* **7**, 46400; doi: 10.1038/srep46400 (2017).

**Publisher's note:** Springer Nature remains neutral with regard to jurisdictional claims in published maps and institutional affiliations.

## Figures and Tables

**Table 1:** Volatile organic compounds for banana plants.

Compounds^a^	CAS	IUPAC names	RIcal^b^	RIStd^c^	RIref^d^	Identification^e^	Class^f^	VOCs quantities ± standard deviation (ng/μl)		Ref RI^g^
								Cavendish	Pacific Plantain	
α-pinene	80-56-8	4,6,6-trimethylbicyclo[3.1.1]hept-3-ene	1031	1018	1032	MS RI STD	Ae	3.30 ± 1.24	—	A
β-pinene	127-91-3	6,6-dimethyl-4-methylidenebicyclo[3.1.1]heptane	1109	1102	1124	MS RI STD	Ae	1.23 ± 0.47	—	A
Myrcene	123-35-3	7-methyl-3-methylideneocta-1,6-diene	1163	1158	1156	MS RI STD	Ae	3.84 ± 1.95	16.89 ± 12.85	A
Limonene	138-86-3	1-methyl-4-prop-1-en-2-ylcyclohexene	1191	1186	1178	MS RI STD	Ae	15.19 ± 5.54	—	B
(E)-hex-2-enal	6728-26-3	(E)-hex-2-enal	1218	1211	1207	MS RI STD	Ad	—	60.23 ± 34.64	A
(Z) β ocimene	3338-55-4	(3Z)-3,7-dimethylocta-1,3,6-triene	1228	1222	1245	MS RI STD	Ae	8.92 ± 4.04	10.36 ± 7.19	B
(E) β ocimene	13877-91-3	(3E)-3,7-dimethylocta-1,3,6-triene	1244	1240	1242	MS RI STD	Ae	8.86 ± 4.45	241.76 ± 158.49	B
4,8-dimethyl-1,3,7-nonatriene	19945-61-0	(3E)-4,8-dimethylnona-1,3,7-triene	1302	—	—	MS	Ae	—	226.20 ± 62.01	
6-methyl-5-hepten-2-one	110-93-0	6-methylhept-5-en-2-one	1328	1329	1340	MS RI STD	K	29.60 ± 11.66	18.56 ± 20.89	D
Alloocimene	673-84-7	(4E,6E)-2,6-dimethylocta-2,4,6-triene	1361	1364	1367	MS RI STD	Ae	—	3.82 ± 2.65	D
Linalool	78-70-6	3,7-dimethylocta-1,6-dien-3-ol	1535	1539	1537	MS RI STD	Ae	—	6.96 ± 2.89	B
6-methyl-3,5-heptadien-2-one	1604-28-0	(3E)-6-methylhepta-3,5-dien-2-one	1576	1583	1590	MS RI STD	K	14.83 ± 10.84	18.78 ± 26.86	E
(E)-β-farnesene	18794-84-8	(6E)-7,11-dimethyl-3-methylidenedodeca-1,6,10-triene	1654	1661	1658	MS RI STD	Ae	—	126.02 ± 87.79	B
(Z,E)-α-farnesene	26560-14-5	(3Z,6E)-3,7,11-trimethyldodeca-1,3,6,10-tetraene	1716	1715	1727	MS RI STD	Ae	—	799.64 ± 503.15	C
(E,E)-α-farnesene	502-61-4	(3E,6E)-3,7,11-trimethyldodeca-1,3,6,10-tetraene	1740	1742	1756	MS RI STD	Ae	87.90 ± 11.28	571.24 ± 381.70	C
Methyl salicylate	119-36-8	methyl 2-hydroxybenzoate	1755	1765	1745	MS RI STD	E	33.82 ± 14.29	145.42 ± 163.03	B
β-ionone	79-77-6	(E)-4-(2,6,6-trimethylcyclohexen-1-yl)but-3-en-2-one	1924	1934	1918	MS RI STD	Ae	3.76 ± 0.85	3.47 ± 1.76	A

^a^Compounds are listed according to their order of elution.

^b^Linear retention index calculated on a VF-Wax capillary column with an homologous series of alkanes.

^c^Linear retention indexes obtained for injected standards.

^d^Linear retention indexes from literature.

^e^Identification methods used are indicated by the following.

MS: identification by comparison with mass spectral databases, RI: identification by retention indexes with literature data, STD: comparison with the retention times and mass spectra of available pure standards; f: Chemical classes: Ae: Alkene, Ad: Aldehyde, E: Ester, K: Ketone; g: References: A: Walter and Shibamoto, 1980, B: http://www.flavornet.org/f_kovats.html, C: Davies, 1990, D: Weingart *et al*., 2012, E: Buttery *et al*., 1990.

**Table 2:** Occurrence of VOCs in plants.

Compounds	Occurrence in plant					
	*Musa sp.* Flower^a^	Fruit of *M.acuminata* cv. Nanicão^b^	Cavendish	Pacific Plantain	Zingiberales excluding *Musa sp.*^*c*^	Other^c^
α-pinene	✓	✓	✓	×	✓	✓
β-pinene	✓	×	✓	×	×	✓
Myrcene	✓	×	✓	✓	✓	✓
Limonene	✓	✓	✓	×	✓	✓
(E)-hex-2-enal	×	×	×	✓	×	✓
(Z) β ocimene	×	×	✓	✓	✓	✓
(E) β ocimene	×	×	✓	✓	✓	✓
4,8-dimethyl-1,3,7-nonatriene	×	×	×	✓	✓	✓
6-methyl-5-hepten-2-one	×	×	✓	✓	✓	✓
Alloocimene	×	×	×	✓	✓	✓
Linalool	×	×	×	✓	✓	✓
6-methyl-3,5-heptadien-2-one	×	×	✓	✓	✓	✓
(E)-β-farnesene	×	×	×	✓	×	✓
(Z,E)-α-farnesene	×	×	×	✓	×	✓
(E,E)-α-farnesene	×	×	✓	✓	✓	✓
Methyl salicylate	×	×	✓	✓	✓	✓
β-ionone	×	×	✓	✓	×	✓

^a^Bestmann *et al*.^11^; ^b^Facundo *et al*.^10^; ^c^Pherobase.com^14^.
